# Host Responses in an *Ex Vivo* Human Skin Model Challenged With *Malassezia sympodialis*

**DOI:** 10.3389/fcimb.2020.561382

**Published:** 2021-01-21

**Authors:** Dora E. Corzo-León, Donna M. MacCallum, Carol A. Munro

**Affiliations:** School of Medicine, Medical Sciences and Nutrition, Institute of Medical Sciences, University of Aberdeen, Aberdeen, United Kingdom

**Keywords:** *Malassezia sympodialis*, skin model, immune response, cytokines, antimicrobial peptides

## Abstract

*Malassezia* species are a major part of the normal mycobiota and colonize mainly sebum-rich skin regions of the body. This group of fungi cause a variety of infections such as pityriasis versicolor, folliculitis, and fungaemia. In particular, *Malassezia sympodialis* and its allergens have been associated with non-infective inflammatory diseases such as seborrheic dermatitis and atopic eczema. The aim of this study was to investigate the host response to *M. sympodialis* on oily skin (supplemented with oleic acid) and non-oily skin using an *ex vivo* human skin model. Host-pathogen interactions were analyzed by SEM, histology, gene expression, immunoassays and dual species proteomics. The skin response to *M. sympodialis* was characterized by increased expression of the genes encoding β-defensin 3 and RNase7, and by high levels of S100 proteins in tissue. Supplementation of oleic acid onto skin was associated with direct contact of yeasts with keratinocytes and epidermal damage. In oily conditions, there was increased expression of *IL18* but no expression of antimicrobial peptide genes in the skin’s response to *M. sympodialis*. In supernatants from inoculated skin plus oleic acid, TNFα, IL-6, and IL1-β levels were decreased and IL-18 levels were significantly increased.

## Introduction

The genus *Malassezia*, previously known as *Pityrosporum*, is a group of lipophilic yeasts. *Malassezia* species are part of the normal mycobiota and colonize several regions of the body, mainly sebum-rich skin areas such as the scalp and thorax (Marcon and Powell, 1992). To date, 17 *Malassezia* species have been proposed (Sparber and LeibundGut-Landmann, 2017). *Malassezia* spp. are the most abundant genus on the skin of individuals with psoriasis and atopic eczema (Paulino et al., 2006; Paulino et al., 2008; Zhang et al., 2011) and are highly increased in seborrheic dermatitis and dandruff (DeAngelis et al., 2005; Park et al., 2012).

*M. globosa*, *M. furfur*, *M. restricta*, and *M. sympodialis* are the most frequent *Malassezia* species on both healthy (Findley et al., 2013) and diseased skin, such as pityriasis versicolor and seborrheic dermatitis (Prohic and Ozegovic, 2007; Prohic, 2010; Amado et al., 2013; Lyakhovitsky et al., 2013; Lian et al., 2014; Rodoplu et al., 2014). *M. sympodialis* is one of the most frequent colonizers of healthy skin (Findley et al., 2013), but is found less frequently in *Malassezia* related-diseases, such as seborrheic dermatitis and pityriasis versicolor (Prohic and Ozegovic, 2007; Prohic, 2010; Amado et al., 2013).

Most *Malassezia* species are unable to synthesize fatty acids and degrade carbohydrates and are dependent upon the acquisition of exogenous fatty acids. *Malassezia* species have a large repertoire of lipolytic enzymes such as lipases, phospholipases and esterases (Saunders et al., 2012; Sparber and LeibundGut-Landmann, 2017). Lipases hydrolyze sebum triglycerides from the host skin to release fatty acids (oleic acid and arachidonic acid). Lipase activity is significantly higher in *M. globosa* and *M. pachydermatis* than in *M. sympodialis* and *M. slooffiae*, although normal phospholipase activity is found in these species (Juntachai et al., 2009). The fatty acids oleic acid (OA) and arachidonic acid are released by phospholipase and lipase activity and have an irritating/inflammatory effect on skin (Ashbee and Evans, 2002). OA is also increased in dandruff scalps compared with non-dandruff scalps (Jourdain et al., 2016) and increases the severity of flaking (or *stratum corneum* desquamation) facilitating better penetration of *M. sympodialis* so that the fungus directly interacts with cells of the inner skin layers (DeAngelis et al., 2005).

Exogenously acquired fatty acids contribute to the formation of a thick cell wall in *Malassezia* sp. characterized by a unique lipid-rich outer layer, which contributes to triggering the immune response against this group of fungi (Thomas et al., 2008; Gioti et al., 2013). Human studies have shown that cytokine levels (IL-1α, IL-1β, IL-2, IL-4, IFN-γ, IL-10, and IL-12) in the skin of individuals with seborrheic dermatitis and *Malassezia* folliculitis were higher than levels in the skin of healthy volunteers (Faergemann et al., 2001). However, this pattern of cytokine induction varied depending on the fungal cell wall structure. Higher levels of IL-8 and lower levels of IL-10 were produced by keratinocytes *in vitro* when they were stimulated with *M. sympodialis* lacking the lipid-rich outer layer compared to the same yeasts with the outer layer (Thomas et al., 2008).

Antimicrobial peptides (AMPs) are key in the innate immune response to *Malassezia* spp. Individuals with pityriasis versicolor have significantly higher AMP levels (β-defensin 2, β-defensin 3, S100A7, and RNase7) in their skin (Brasch et al., 2014). The β-defensins are specifically increased in the *stratum corneum*, RNase7 in the *stratum granulosum*, and S100A7 in the *stratum corneum, granulosum* and *spinosum* (Brasch et al., 2014). *Malassezia* sp. also play a role in NLRP3 inflammasome activation when yeast cells are sensed by dendritic cells but not by keratinocytes (Kistowska et al., 2014). Activation of NLRP3 depends on Dectin-1 and leads to high expression of caspase-1-dependent IL-1β in dendritic cells of patients with seborrheic dermatitis (Kistowska et al., 2014). The adaptive immune response against *Malassezia* is characterized by the production of specific IgG and IgM antibodies in healthy individuals and specific IgE antibodies in atopic eczema (Glatz et al., 2015).

Atopic eczema (AE) is a chronic inflammatory disease affecting up to 20% of children and 3% of adults (Nutten, 2015). Multiple factors have been associated with AE, such as impairment of skin barrier function due to physical (scratching, skin dryness) or chemical damage (pH changes due to soap), genetic factors (mutations in *FLG* and *SPINK5* genes encoding filaggrin and serine protease inhibitor Kazal-type 5 protein, respectively), and environmental factors (cold climate, no breastfeeding, pollution) (Sääf et al., 2008; Nutten, 2015). *Malassezia* sp. has been linked with AE pathogenesis as *Malassezia* sp. allergens induce specific IgE antibodies and autoreactive T-cells that can cross-react with skin cells (Zargari et al., 2001; Glatz et al., 2015).

The *M. sympodialis* genome contains 13 allergen genes, Mala S1, Mala S5 to S13 and three orthologs of *M. furfur* allergens (Mala F2, 3, and 4) (Andersson et al., 2003; Gioti et al., 2013). Some of these allergens are highly similar to human proteins (Mala S11, Mala S13) and have been specifically linked to cross-reactive immune responses (Schmid-Grendelmeier et al., 2005; Gioti et al., 2013; Roesner et al., 2019).

The aim of this study was to investigate the host skin response to *Malassezia sympodialis.* An *ex vivo* human skin model was used either directly for non-oily skin or supplemented with oleic acid to represent oily skin, and the skin surface was scratched to represent a disrupted skin barrier. Host-pathogen interactions were analyzed by SEM, histology, gene expression, immunoassays, and proteomics.

The skin response to *M. sympodialis* was characterized by increased expression of the genes encoding β-defensin 3, and RNase7 and high levels of S100 proteins. Supplementation of the skin with oleic acid resulted in epidermal damage, direct contact of yeasts with keratinocytes, and AMP gene expression was not detected. IL-18 levels were significantly increased in supernatants from inoculated skin plus oleic acid and *IL18* gene expression was increased in tissue. TNFα, IL-6, and IL-1β levels were decreased in the same supernatants.

## Materials and Methods

### Fungal Strains and Culture Conditions

Modified Dixon (mDixon) broth and agar (3.6% w/v malt extract, 2% w/v desiccated ox-bile, 0.6% w/v bacto tryptone, 0.2% v/v oleic acid, 1% v/v Tween 40, 0.2% v/v glycerol, 2% w/v bacto agar for agar plates) was used to grow *Malassezia sympodialis* (ATCC 42132) for skin experiments. Yeast cells were grown on mDixon agar at 35°C for 4–5 days in a static incubator, one colony was selected from an agar plate and inoculated into 10 ml of mDixon broth for 4 days at 37°C in a shaking incubator at 200 rpm. Yeast cells were recovered and a final inoculum of 1x10^6^ yeasts in 10 µl of PBS (1x10^8^/ml) was prepared and applied to the skin surface.

### An *Ex Vivo* Human Skin Model

The *ex vivo* human skin model was set up as previously described with modifications (Corzo-León et al., 2019). Human skin tissue (no stretch marks), without an adipose layer (adipose layer removed by the surgeon during surgery), from abdominal or breast surgeries was supplied by Tissue Solutions^®^ Ltd. (Glasgow, UK). Human tissue from four different donors was obtained according to the legal and ethical requirements of the country of collection, with ethical approval and anonymous consent of the donor or nearest relative. Tissue Solutions^®^ comply with the UK Human Tissue Authority (HTA) on the importation of tissues. Explants were transported at 4°C with cooling packs and maintained at 4°C until processed, which occurred within 36 h of surgery.

Skin was washed with DMEM (supplemented with 1% v/v antibiotics (penicillin and streptomycin) and 10% heat inactivated FBS, Thermo-Fisher Scientific, Loughborough, UK) and kept moist in a Petri dish in the same medium. The explant was cut into 1 cm^2^ pieces. The surface of each piece was gently wounded using a needle, without penetrating the entire skin thickness, by pricking with a needle several times (6-10) to a depth of approximately 2–3 mm.

After wounding, each piece of skin was placed into an individual well of a 6-well plate. An air-liquid interphase was maintained by adding 1 ml of supplemented DMEM. A well containing only DMEM growth medium was added as a negative control and served as a contamination control. The medium was changed every 24 h and the spent medium was stored in 2 ml tubes for subsequent analysis at the same time points as the rest of the samples. Recovered medium was stored at -80°C until analyzed. In a previous study (Corzo-León et al., 2019), the skin samples were demonstrated to remain viable for 14 days under non-oily conditions using the TUNEL system (Promega, Southampton, UK). This same assay was used to evaluate cell viability and apoptosis in 6 µm skin tissue sections from the frozen OCT blocks in this study. Apoptosis levels were measured in the four groups of skin samples in three biological replicates which were analyzed in duplicate. Apoptosis levels were averaged, statistical analysis was done using one-way ANOVA test. For post-hoc analysis, Tukey’s test was used. Prism 8 software (GraphPad, La Jolla, CA, USA).

Skin explants were inoculated by applying 10 µl of fungal suspension (1 x 10^6^ yeasts) directly on to the epidermis. Yeast suspension was prepared as described below and resuspended in PBS. Uninfected/non-inoculated skin controls were included in all experiments. Two additional experimental conditions were added: 1) uninfected skin with 10 µl 100% oleic acid (Oleic Acid, Extra Pure, catalogue number: O/0200/15, Thermo-Fisher Scientific) applied to the surface of the skin explant and 2) infected skin with same *M. sympodialis* inoculum, followed by application of 10 µl 100% oleic acid on to the surface of the skin explant.

Skin samples were incubated for 6 days at 37°C and 5% CO_2_ before being recovered in a Petri dish. Prior to processing, the macroscopic appearance of explants was evaluated by eye and images captured with a Stemi 2000-c Stereo Microscope (Carl Zeiss, Oberkochen, Germany). Samples were then processed for further analyses.

Tissue samples for histology were placed into molds, embedded in OCT compound (Cellpath Ltd. Newtown, UK) and flash-frozen with dry ice and isopentane. These samples were stored at -20°C for immediate analysis or at -80°C for longer term storage. For scanning electron microscopy (SEM), tissue samples were fixed in glutaraldehyde buffer (2.5% glutaraldehyde in 0.1 M cacodylate) overnight at 4°C and sent to the Microscopy and Histology technology hub, University of Aberdeen, for further sample preparation. Tissue samples for RNA extraction were cut into smaller pieces and placed in a microcentrifuge tube with RNAlater^®^ (Sigma, Dorset UK) for subsequent RNA extraction. These samples were stored at -20°C for immediate analysis or at -80°C for longer term storage. Experiments were replicated at least three times using skin from different human donors.

### Scanning Electron Microscopy and Histopathology

Several microscopy analyses were performed on recovered skin tissue for histological confirmation of fungal infection. Sections (6 µm) were cut from the frozen OCT blocks for histological analysis and stained with fluorescent dyes (1 µg/ml calcofluor white (CFW), and propidium iodide (1 µg/ml). Tissue sections were also stained with Periodic acid solution (1%), Schiff reagent and counterstained with Hematoxylin solution (Sigma). Fluorescent images were captured with a DeltaVision™ confocal microscope (GE Healthcare, Buckinghamshire UK). PAS histological sections were imaged by light microscopy using a Zeiss™ Axioskop microscope. SEM samples were observed using a Zeiss™ EVO MA10 Scanning Electron Microscope. Images were generated by detection of secondary electrons (SE1) and backscatter electron detection (NTS BSD) and captured at 10 kV resolution and at different magnifications.

### TUNEL Analysis to Measure Cell Viability

The percentage of apoptotic cells was determined by counting the apoptotic cells (green cells stained by fluorescein-12-dUTP) and expressing them as a percentage of the total number of cells (apoptotic cells and non-apoptotic cells stained red with propidium iodide). Three biological replicates were analyzed for each experimental condition with duplicate slides prepared for each replicate. Three fields were analyzed per slide with a total of at least 600 cells counted for each biological replicate.

### Gene Expression and Proteomics Analyses

Tissue samples for RNA extraction were thawed and the RNAlater^®^ discarded before processing the samples. Samples were washed twice with PBS. RNA and proteins were extracted from the same recovered samples in a single 2-day sequential process based on previously published methods (Chomczynski and Sacchi, 1987; Berglund et al., 2007; Corzo-León et al., 2019).

The RNA yield and purity were evaluated by Nanodrop™ spectrophotometry (Thermo-Fisher Scientific) and samples were stored at -80°C until further use. All samples had an initial yield between 200 and 800 ng/µl and a 260/280 ratio between 1.8 and 2.0. To produce cDNA, RNA samples (1 µg) were treated with DNase I (1 U/µl per 1 µg of RNA sample) (Thermo-Fisher Scientific), then reverse transcription carried out using the SuperScript™ IV first-strand synthesis system (Thermo-Fisher Scientific) with Oligo dT primer, following the manufacturer’s instructions.

Intron spanning primers and qRT-PCR assays were designed using Roche’s Universal Probe Library Assay Design Centre (lifescience.roche.com/en_gb/brands/universal-probe-library.html#assay-design-center) for different target genes known to be expressed in skin. Genes, accession numbers, Roche probes paired with target primers and primer sequences are shown in **Table 1**. The Roche probes were hydrolysis probes labelled at the 5ʹ end with fluorescein (FAM) and at the 3ʹ end with a dark quencher dye. A reference gene (*B2M* encoding β2-microglobulin) primer pair and probe was also designed (Lossos et al., 2003) using the Eurogentec web tool (secure.eurogentec.com/life-science.html) (**Table 1**). The probe was modified at the 5ʹ end with Cy5 and at the 3ʹ end with quencher QXL 670.

**Table 1:** Primers targeting human genes.

Target Gene/Accession number	Primer	Sequence (5ʹ-3ʹ)
TGFB1 NM_000660.6	Primer F	TGGACATCAACGGGTTCAC
	Primer R	GGCCATGAGAAGCAGGAA
	Roche hydrolysis Probe^++^	#49
IL18 NM_001243211.1	Primer F	CAACAAACTATTTGTCGCAGGA
	Primer R	CAAAGTAATCTGATTCCAGGTTTTC
	Roche hydrolysis Probe^++^	#66
RNASE7 NM_032572.3	Primer F	CAGGAGTCACAGCACGAAGA
	Primer R	CAGCAGAAGCAGCAGAAGG
	Roche hydrolysis Probe^++^	#15
S100A8/ NM_001319196.1	Primer F	CAGCTGTCTTTCAGAAGACCTG
	Primer R	TGTGGTAGACGTCGATGATAGAG
	Roche hydrolysis Probe^++^	#78
S100A7 NM_002963.3	Primer F	CCAAACACACACATCTCACTCA
	Primer R	TCAGCTTGAGTGTTGCTCATC
	Roche hydrolysis Probe^++^	#33
S100A9 NM_002965.3	Primer F	GTGCGAAAAGATCTGCAAAA
	Primer R	TCAGCTGCTTGTCTGCATTT
	Roche hydrolysis Probe^++^	#85
DEFB4A NM_004942.3	Primer F	TCAGCCATGAGGGTCTTGTA
	Primer R	AGGATCGCCTATACCACCAA
	Roche hydrolysis Probe^++^	#35
DEFB103B NM_018661.4	Primer F	TTCTGTTTGCTTTGCTCTTCC
	Primer R	CGCCTCTGACTCTGCAATAA
	Roche hydrolysis Probe^++^	#85
	**REFERENCE GENE**	**Sequence**
β2-microglobulin NM_004048.2	β2M Primer F*	TGACTTTGTCACAGCCCAAGATA
	β2M Primer R*	CGGCATCTTCAAACCTCCA
	Probe**	ACATGTCTCGATCCCAC

^++^Primers and matching probes were selected using Universal Probe Library Assay Design center. All Roche probes were hydrolysis probes labelled at the 5ʹ end with fluorescein (FAM) and at the 3ʹ end with a dark quencher dye. *Primers were designed to amplify the β2-microglobulin gene, which was used as the reference gene **Modified probe at the 3ʹ end with QXL 670, and at the 5ʹ end with Cy5.

qRT-PCR reactions (10 µl) were set up in Light cycler 480 plates using the LightCycler 480 probe master mix (Roche, Welwyn Garden City UK) according to the manufacturer’s instructions (**Table 2**). Dual hydrolysis probe assays were analyzed in the same well, FAM probe was used for target gene primer pairs and Cy5 probe for the reference gene. For each cDNA, assays were performed in triplicate.

**Table 2:** qRT-PCR reactions for skin samples.

Reagent	Volume (µl)	Final concentration (nM)
Master mix (Roche)	5.0	
10 µM Target primer forward	0.25	250
10 µM Target primer reverse	0.25	250
1 µM Target probe (FAM)	0.5	50
10 µM Reference primer forward	0.25	250
10 µM Reference primer reverse	0.25	250
1 µM Reference probe (Cy5)**	0.25	50
Water	1.25	–
cDNA	2.0	150–200 ng/µl
**Total volume**	**10 µl**	

For skin samples, dual hydrolysis probe assays were analyzed in the same well, with a FAM probe used for target genes primer pairs and a Cy5 probe for the reference gene.

Reactions were run in a LightCycler 480 (Roche). Following manufacturer’s recommendations, reaction settings were as follows: one cycle at 95°C for 10 min (ramp rate 4.8°C/s), 55 cycles of amplification phase with denaturation at 95°C for 10 s (ramp rate 4.8°C/s), annealing at 60°C for 30 s (ramp rate 2.5°C/s), extension 72°C for 1 s (ramp rate 4.8°C/s); and, finally, one cycle of cooling phase at 40°C for 30 s (ramp rate 2.5°C/s). Results obtained for each target gene were normalized against β2-microglobulin gene expression levels. The corresponding uninfected/non-inoculated skin samples (with or without OA) were used as negative controls for infection and to measure baseline gene expression levels. Results were analyzed using the 2-ΔΔCT method (Livak and Schmittgen, 2001). Statistical analysis was performed using the Student’s t-test or Mann-Whitney test depending on the data distribution using Prism 8 software (GraphPad, La Jolla, CA, USA).

Protein concentration was determined by Coomassie G-250 Bradford protein assay kit following manufacturer’s instructions (Thermo-Fisher Scientific). Protein samples were sent for trypsin digestion and LC-MS/MS analysis (Aberdeen Proteomics Core Facility (www.abdn.ac.uk/ims/facilities/proteomics/). Four biological replicates were analyzed per condition (infected and uninfected skin). Only proteins having two or more identified peptides and two or more Peptide Spectrum Matches (PSM) were selected. Finally, proteins found in at least two out of four analyzed samples per condition were included for further Gene Ontology (GO) analysis using the GO consortium online tool (geneontology.org). Area under the curve (AUC) values for each protein were averaged and compared between conditions, inoculated and non-inoculated skin, non-inoculated skin with and without oleic acid and, finally analyzed using the Student’s t-test or Mann-Whitney test depending on the distribution of the data, with a value of p<0.05 considered statistically significant (Prism 8 software). Proteins identified as significant were compared to the CRAPome database (www.crapome.org/). The CRAPome web tool is a Contaminant Repository for Affinity Purification and contains lists of proteins identified in negative control samples, collected using affinity purification followed by mass spectrometry (AP-MS). Proteins found in the CRAPome database in >10% of the cases were not considered significant as they are probably the result of carryover contamination during mass spectrometry experiments.

### Immunoassays

Recovered supernatants from inoculated skin and non-inoculated skin controls were analyzed for different cytokines (TGFβ1, TNFα, IL-1β, IL-6, IL-8, IL-18, IFNγ, and TNFRI) at 2–3 days of incubation. Four biological experiments were analyzed in duplicate. Multiplex immunoassays were carried out following the manufacturer’s instructions (Milliplex ^®^ Map kits. EMD Millipore Corporation, Livingston UK). Data were analyzed using either one-way ANOVA or Kruskal-Wallis test depending on the homogeneity of variance (tested by Bartlett’s test). A *p* value <0.05 was considered statistically significant (Bonferroni correction) and post-hoc analysis done by Dunnett’s or Dunn’s test (Graphpad Prism 8).

## Results

### *M. sympodialis* Invaded *Ex Vivo* Skin Supplemented With Oleic Acid and Interacted Directly With the Inner Epidermal Layer

Host-pathogen interactions between human skin and *M. sympodialis* were investigated using an explant human skin model with skin collected from four healthy donors undergoing cosmetic surgeries (Corzo-León et al., 2019). All skin samples were gently wounded by scratching the surface with a needle. Four different skin conditions were used: untreated skin left uninfected or inoculated with *M. sympodialis* (see below) or skin supplemented with 10 µl of 100% oleic acid (OA) and uninfected or inoculated with *M. sympodialis* (MS). Inoculated skin samples were inoculated with 1x10^6^ *M. sympodialis* yeast cells in 10 µl applied onto the skin surface.

**Figure 1:**
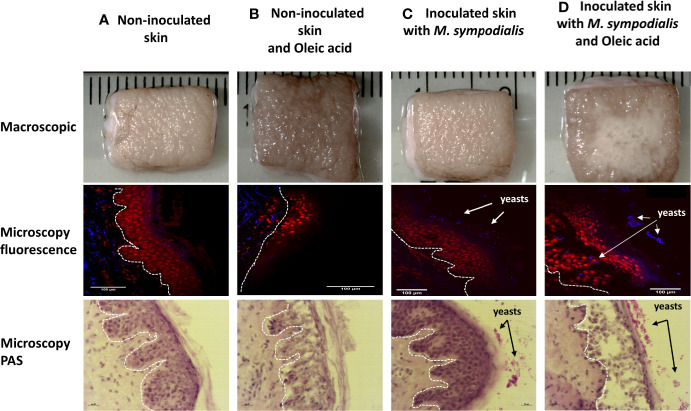
Macroscopic and histologic appearance of skin in four different conditions. Human skin explant samples were untreated **(A, C)** or supplemented with oleic acid **(B, D)**. *M. sympodialis* (1 x 10^6^ yeasts) was inoculated in **(C, D)**, with **(A, B)** uninoculated. All samples were incubated at 37°C in 5% CO_2_ for 6 days. Scale is indicated by the ruler, with the space between each bar = 1 mm. Fluorescence microscopy images show propidium iodide (PI) (red) indicating keratinocytes in epidermis and CFW (blue) staining *M. sympodialis* yeast cells after 6 days incubation. Channels used were DAPI (358 nm/461 nm) for CFW, and Rh-TRITC (543 nm/569 nm) for PI. Scale bars represent 100 µm. PAS staining images are shown on the bottom row. Scale bars represent 20 µm. In all histological samples, the white line separates the dermis from epidermis. Epidermis in all images are facing right side. Yeasts are seen on the external side of the skin. Images are representative of four biological replicates per condition.

Skin inoculated with yeasts without OA supplement did not show macroscopic differences when compared to the non-inoculated skin control after 6 days. The inoculated skin did not show any macroscopic changes and will be referred to as inoculated skin instead of infected skin. Meanwhile, skin inoculated with yeasts and supplemented with OA had visible yeast growth (**Figure 1**).

**Figure 2:**
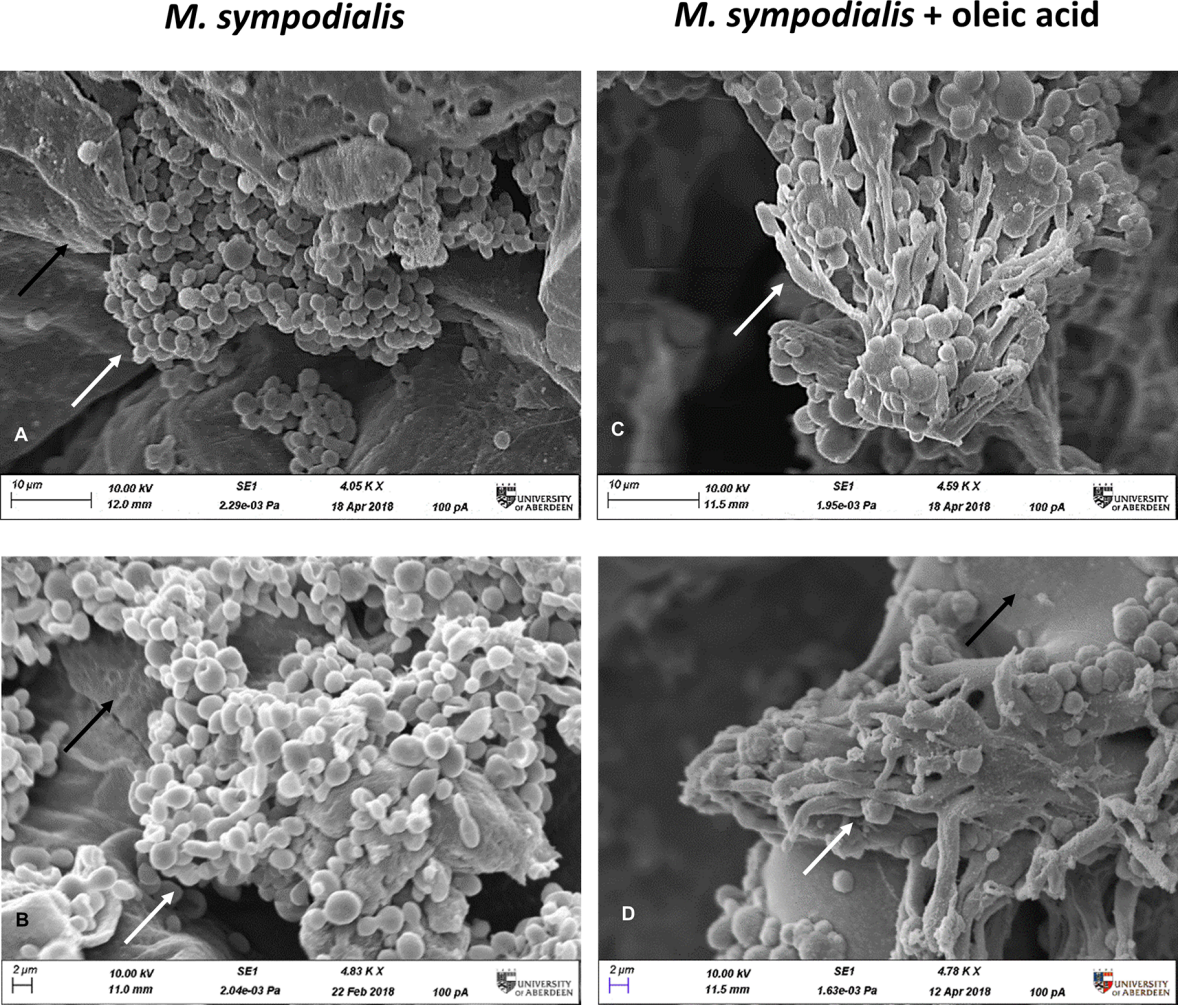
Microscopic appearance of skin inoculated with *M. sympodialis*. *M. sympodialis* yeast cells (1 x 10^6^) were inoculated onto the human skin explants, with or without OA supplementation. All samples were incubated at 37⁰C in 5% CO_2_ for 6 days. **(A, B)** SEM images *M. sympodialis*-inoculated skin, **(C, D)** skin co-inoculated with *M. sympodialis* and oleic acid. Black arrows indicate corneocytes (skin), white arrows indicate fungal structures. Scale bars represent 10 µm **(A, C)** or 2 µm **(B, D)**. Images are representative of four biological replicates.

When analyzed microscopically by SEM, only the *M. sympodialis*-inoculated skin had fungal structures on the epidermis. The fungal structures differed between the skin supplemented with OA and skin that did not receive OA. Skin without OA (MS or *Malassezia* only) had yeast cells on the top of the skin, while skin receiving both yeasts and OA (MSOA) had not only more abundant yeasts but also elongated fungal structures (**Figure 2**).

Tissue sections were stained with propidium iodide to stain nuclei in the epidermis and calcofluor white (CFW) to stain chitin in the yeast cell walls. In parallel, sections were stained with PAS. The sections were examined to investigate the integrity of keratinocytes and presence of fungal elements. The epidermis in different samples had differences in structure. Sections of MSOA skin had detached keratinocyte layers and had yeast cells in direct contact with the inner epidermal layers. In contrast, MS skin had completely intact epidermis and yeast cells were trapped in the outer *stratum corneum* layer. Intact epidermis was also seen in the uninfected skin control without oleic acid. In uninfected skin receiving OA, thinner epidermis and damaged keratinocytes were observed, but no detachment of epidermal layers was seen (**Figure 1**).

**Figure 3:**
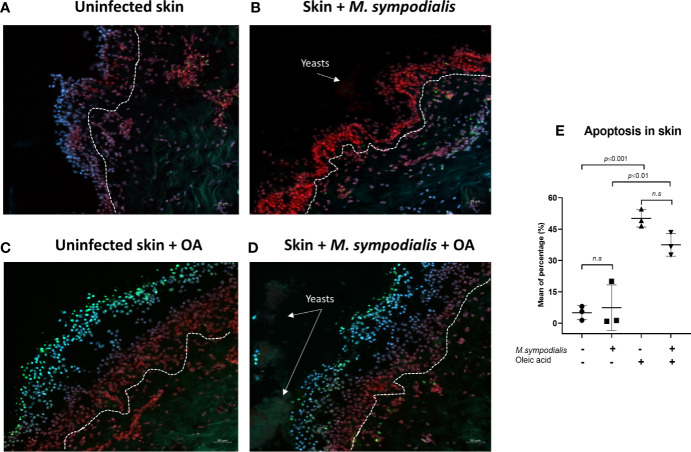
Apoptosis in skin. Apoptosis levels were measured in the four groups of skin samples after 6 days of incubation (images **A–D**). Skin sections were stained with TUNEL system, DAPI and propidium iodide. Apoptotic cells were green, non-apoptotic cells were red/blue/purple (merging of DAPI and Propidium iodide). Channels used were FITC (495 nm/519 nm), DAPI (358 nm/461 nm), Rh-TRITC (543 nm/569 nm). **(E)** Mean apoptosis levels and SD. One-way ANOVA test was applied for statistical analysis (p ≤ 0.0001), post-hoc analysis used the Tukey’s test with *p* values shown in figure, n= 3 biological replicates, with each sample analyzed in duplicate. Scale bars are 50 µm.

When cell viability was evaluated by the TUNEL system, no differences were found between the uninfected skin conditions and their corresponding infected counterparts (**Figure 3**). Cell viability was affected by OA as skin samples treated with OA (either infected or uninfected) had higher numbers of cells showing apoptosis compared to untreated samples (**Figure 3**).

### Local Skin Response to *M. sympodialis* Is Characterized by Higher Expression of Genes Encoding β-Defensin 3, Ribonuclease 7, and Higher Levels of S100 Proteins

**Figure 4:**
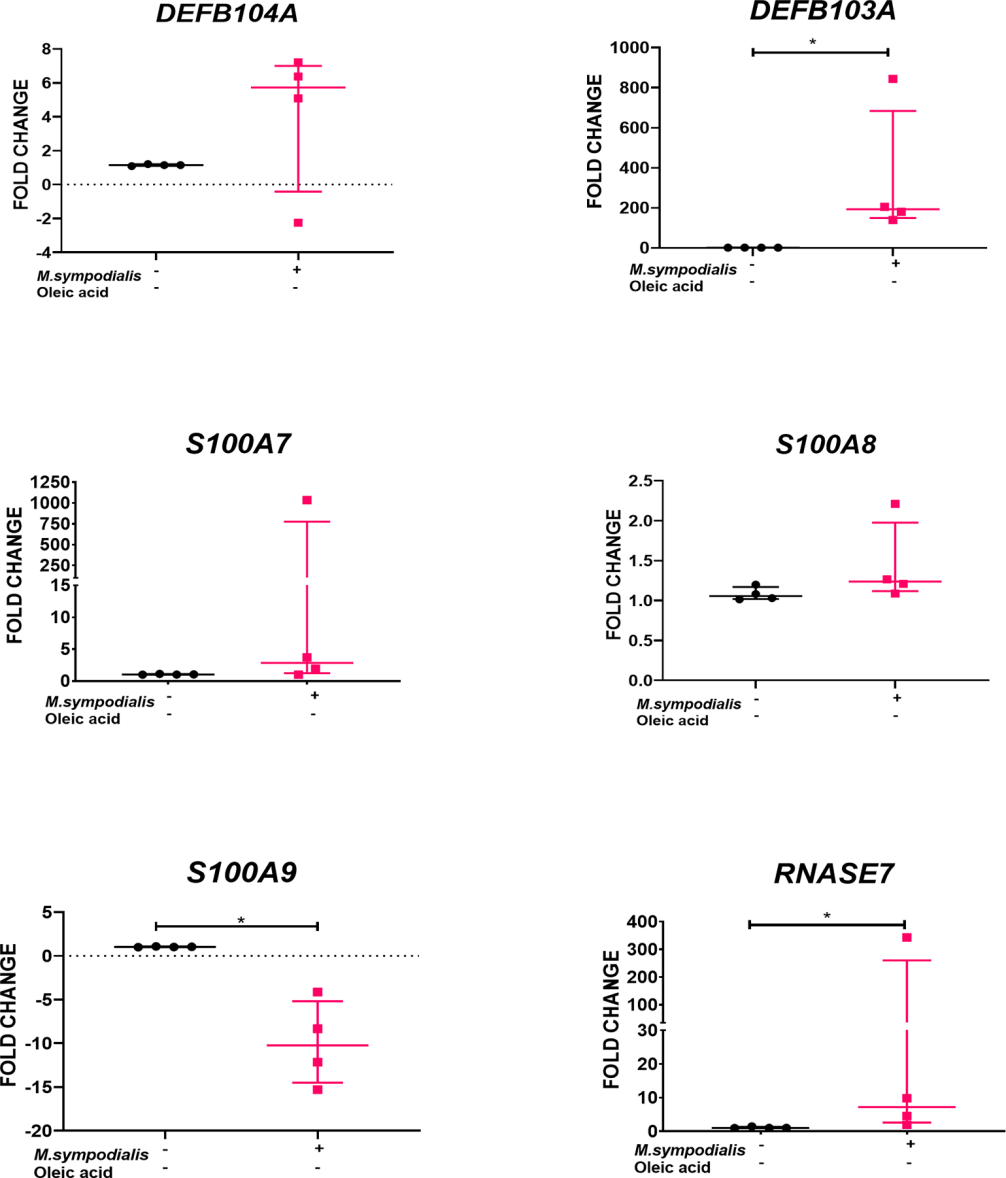
AMP gene expression in *M. sympodialis* infected human skin without OA. RNA was extracted from skin samples after 6 days incubation for qRT-PCR analysis. Results obtained for each target gene were normalized against β2-microglobulin gene expression levels and expressed relative to uninfected skin negative controls. Data is shown as median and IQR and compared by Mann-Whitney U test. n=4 biological replicates, each analyzed in triplicate. *p value = ≤0.05.

RNA was extracted from skin tissue samples for gene expression analysis, at day 6 post-inoculation under the four experimental conditions described above. Due to the importance of AMPs and cytokines in the innate immune response, the expression of eight human genes encoding different AMPs and cytokines was analyzed by qRT-PCR. AMP genes included *S100A7* (psoriasin), *S100A8*, *S100A9*, *DEFB4A* (β-defensin 2), *DEFB103A* (β-defensin 3), *RNASE7* (ribonuclease 7, RNase7), *IL18*, and *TGFB1* (transforming growth factor β1). Expression of AMP genes was found only in uninfected skin and MS skin (inoculated skin without OA). No expression of AMP genes was found in samples receiving OA. We can confirm that reference genes were amplified and expressed in samples receiving OA verifying that the lack of expression of AMP genes was not due to a technical problem. In MS skin, expression of *DEFB103A* (*p ≤* 0.03) and *RNASE7* (*p ≤* 0.03) was increased 193 (IQR 139 to 843) and 7 (IQR 2 to 343) times, respectively, compared to non-inoculated, negative control skin (**Figure 4**). Meanwhile, *S100A9* was significantly (*p ≤* 0.03) decreased -10.2 (IQR -15 to -4) fold.

The four different experimental conditions (described above) were also analyzed by proteomics at 6 days post-infection to gain a better understanding of the host response to oleic acid and *M. sympodialis* and to investigate the *M. sympodialis* proteome when it was interacting with human skin. Trypsin digestion and protein identification by LC-MS/MS was performed by the Aberdeen Proteomics Facility (data in **Supplementary Material S1**, **S2**). Database searches were conducted with the Mascot server v 2.5 using *Homo sapiens* and *M. sympodialis* protein sequences (Swiss-Prot database). Data are available *via* ProteomeXchange with identifier PXD018404.

A total of 1488 proteins were found in skin tissue of the four biological experiments at the 6-day time point. After screening for proteins having ≥2 PSM and ≥2 peptides, the total number of proteins included in the analysis was 1048. Non-inoculated skin conditions (with and without OA) were compared. Most of the proteins (98%) found in the non-inoculated skin receiving OA had significantly decreased levels (-2 fold change) when compared to proteins in the non-OA non-inoculated skin control. Fifteen proteins were significantly decreased (*p ≤* 0.05) in non-inoculated skin receiving OA compared with the non-OA control from which five are involved in cornification, keratinocyte differentiation and wound healing (KRT5, KRT6A, KRT6B, KRT6C, KRT84). (**Supplementary Material S3**).

**Figure 5:**
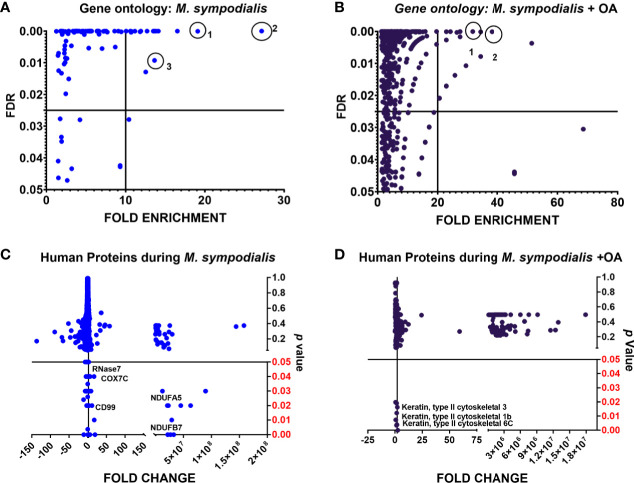
Analysis of human proteins in skin tissue inoculated with *M. sympodialis*. Human proteins with higher levels in tissue at day 6 post-infection (≥2 fold change compared to their corresponding non-inoculated control: non-OA, OA) were analyzed by gene ontology. Statistical analysis was done by Fisher’s test with FDR correction (*p* value). **(A)** biological processes in *M. sympodialis*-inoculated skin, circles are 1) antimicrobial response, 2) cornification 3) defense response to fungus. **(B)** processes in skin co-inoculated with *M. sympodialis* and oleic acid, circles are 1) removal of superoxide radicals, 2) chylomicron remodeling. **(C, D)** show proteins with significantly increased levels in each condition. Fold change was estimated relative to protein level in non-inoculated skin without OA.

Compared to non-inoculated skin, 267/1488 (18%) proteins were greatly increased in the MS condition, while 376/1488 (25%) proteins were greatly increased in the MSOA samples compared to non-inoculated skin that received oleic acid only. Proteins with higher levels compared to their corresponding negative control (± OA) were analyzed by gene ontology. The most significantly enhanced biological processes in MS skin were cornification (fold change 27, FDR <0.0001), antimicrobial immune response (fold change 16.5, FDR <0.0001), and defense response to fungus (fold change 14, FDR 0.009). Meanwhile, for MSOA, chylomicron remodeling (fold change 38, FDR <0.0001) and removal of superoxide radicals (fold change 32, FDR <0.0001) were the most significantly increased processes (**Figure 5**). Keratins were significantly more abundant in MSOA, compared to the plus OA uninfected control and included the five keratins that were less abundant in the plus OA non-inoculated control when compared to non-inoculated control without oleic acid, mentioned above (**Figure 5**, **Supplementary Material S3**).

**Figure 6:**
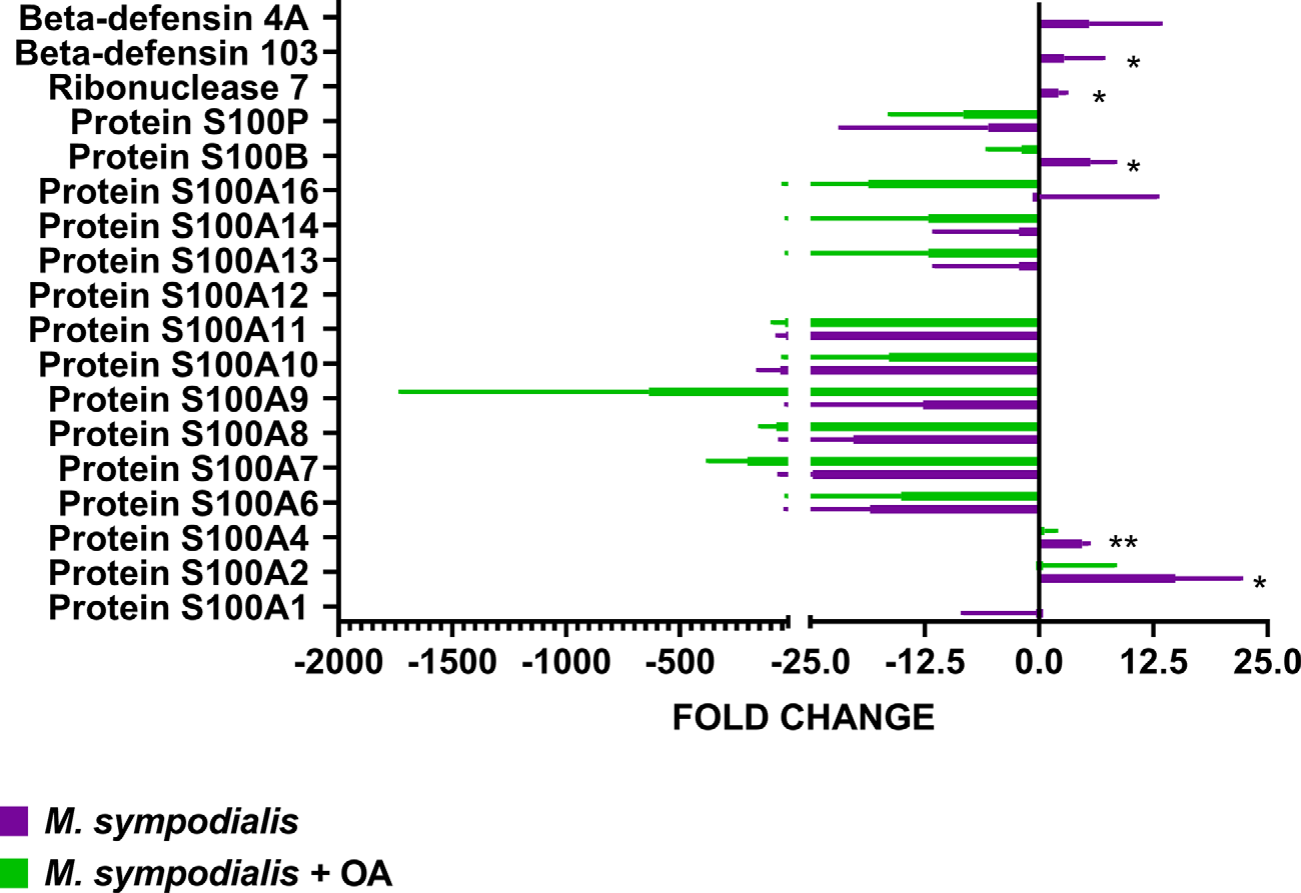
Proteomic analysis of AMP response in skin inoculated with *M. sympodialis*. Single proteins were analyzed separately by Kruskal-Wallis test. If *p* value <0.05 after Kruskal-Wallis then post-hoc analysis by Dunn’s test was performed and indicated in the graphic as *p ≤ 0.05, **p ≤ 0.001. Data are presented as median and IQR, n=4 biological replicates.

AMP levels were analyzed separately and compared to non-inoculated control by Kruskal-Wallis test and Dunn’s test, as well as using the CRAPome online tool. RNase7 (fold change 2, IQR 1–3, CRAPome 0%, *p*=0.05), S100B (fold change 5, IQR 3-9, CRAPome 0, *p*=0.03), S100A4 (fold change 5, IQR 4-5, CRAPome 2%, *p*=0.01), and S100A2 (fold change 14, IQR 7–24, CRAPome 1%, p=0.05) were significantly increased in the MS skin but not in MSOA skin, where they were either decreased or undetectable (**Figure 6**). Along with these AMPs, CD99/MIC2, a glycosylated transmembrane protein (fold change 2, IQR 1.7–3.6, *p*=0.05, CRAPome 1%) was also found at higher levels in the MS skin (**Figure 6**).

### Secreted Cytokine Responses of Human Skin Tissue Exposed to *M. sympodialis* Is Characterized by High Levels of TGFB1 and IL-18

**Figure 7:**
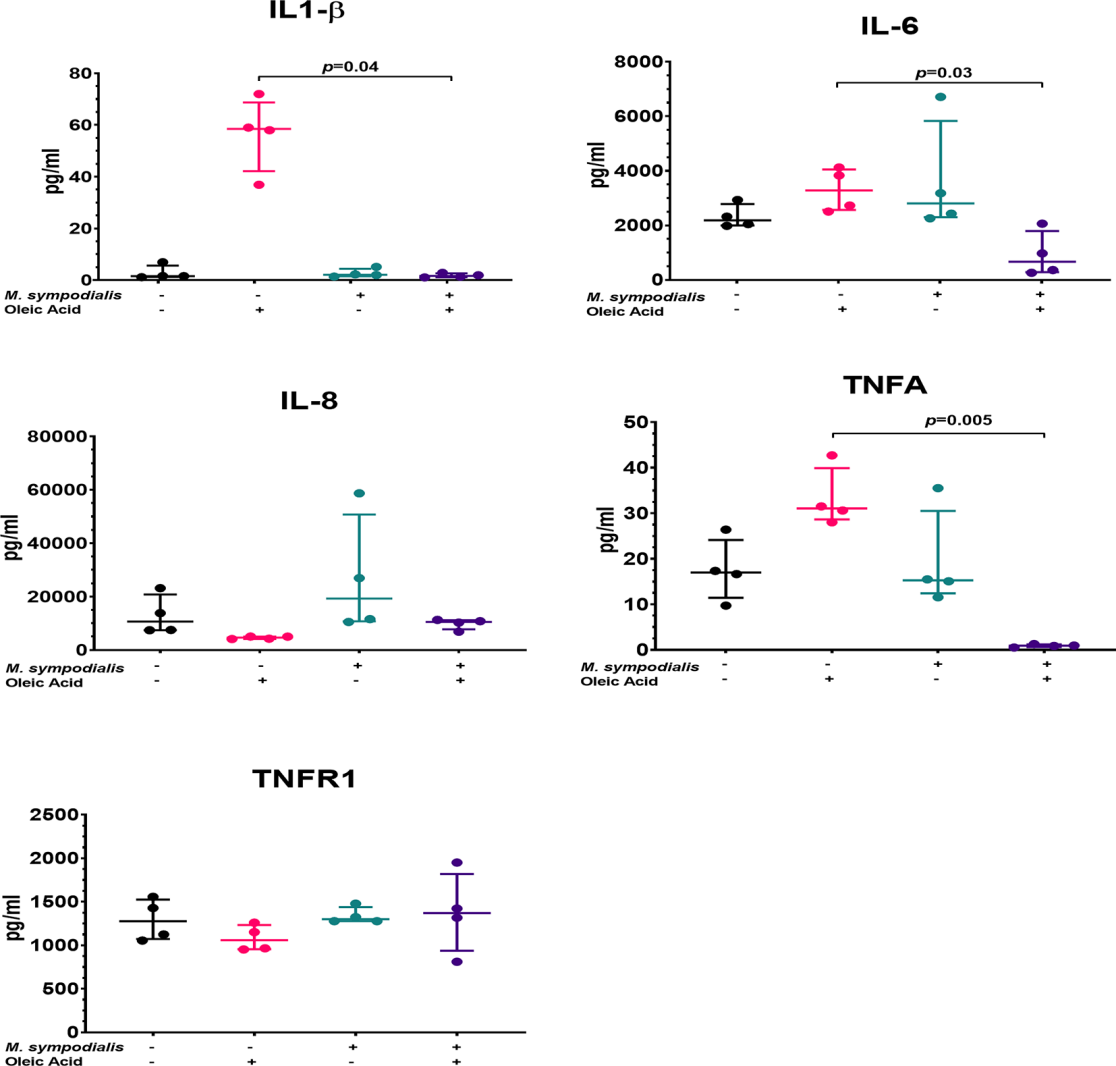
Supernatant cytokine levels from *M. sympodialis*-inoculated skin at 2 days incubation. Cytokine levels were measured by immunoassay. Fold change was calculated by comparing levels to negative control, non-inoculated skin. Data is shown as median and IQR, and analyzed by Kruskal-Wallis test. If *p* value <0.05 after Kruskal-Wallis test then post-hoc analysis by Dunn’s test was performed and indicated in the graphic. n=4 biological replicates, each analyzed in triplicate.

After 2 days of incubation supernatants were recovered from the four experimental conditions and analyzed for seven different cytokines (IL1-β, IL-6, IL-8, TNFR1, TNFα, TGF1β, IL-18). Post-hoc analysis indicated no differences in IL-8 and TNFR1 levels, while IL1-β levels were significantly increased in skin treated with OA compared to MSOA skin (*p*=0.04) (**Figure 7**). IL-6 were significantly higher (*p*=0.03) in OA supernatants compared to MSOA supernatants, but neither levels were significantly different to the non-inoculated control. TNFα levels were not different between non-inoculated control and MS skin but were significantly decreased in MSOA skin compared to OA skin (*p*=0.005) (**Figure 7**).

**Figure 8:**
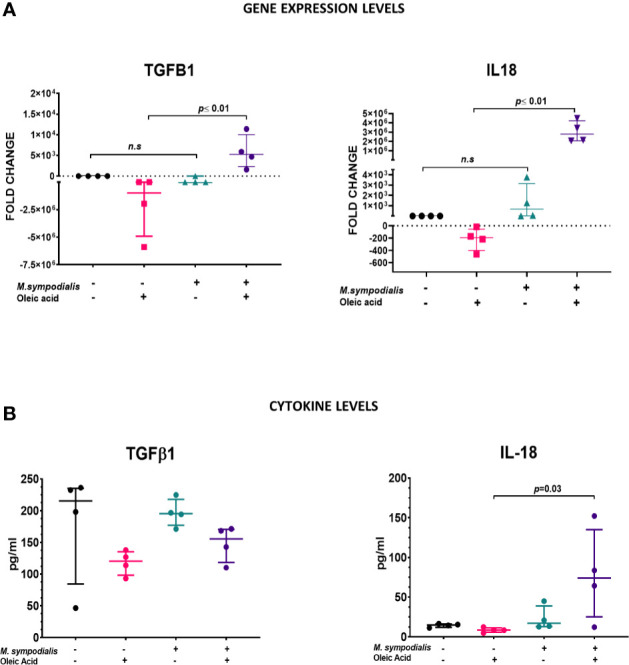
Comparison of TGFB1 and IL-18 expression in tissue and supernatants. **(A)** For gene expression, relative quantification was performed by qRT-PCR using RNA extracted from samples at 6 days post inoculation. Results obtained for each target gene were normalized against β2-microglobulin gene expression levels. Fold change was estimated relative to expression in non-inoculated skin. **(B)** Protein levels in supernatant at 2 days post inoculation were measured by immunoassay and expressed as pg/ml. Data is shown as median and IQR and statistical analysis was done with Kruskal-Wallis test. If p value <0.05 after Kruskal-Wallis test, then post-hoc analysis by Dunn’s test was performed and this value it is indicated in the graphic. n=4 biological replicates, each analyzed in triplicate.

To further explore the role of IL-18 and TGF β1 a more detailed analysis of these two cytokines in tissue and supernatant samples was performed. Gene expression of *TGFB1* and *IL18* in tissue was measured by qPCR and expressed as fold changes compared to uninfected control. *TGFB1* and *IL18* were both increased in MSOA skin compared to oleic acid uninfected skin levels (*p ≤* 0.01). *TGFB1* had a fold change of 5.2 x 10^3^, (IQR 1.6 x 10^3^ to 1 x 10^4^) in MSOA versus a fold change of -9.8 x 10^5^, (IQR -4.9 x 10^6^ to -1.5 x 10^2^) in oleic acid uninfected skin. *IL18* had a fold change of 2.8 x 10^6^, (IQR 2 x 10^6^ to 4.2 x 10^6^) in MSOA versus a fold change of 1.9 x 10^2^ (IQR -4.0 x 10^2^ to -56.6) in oleic acid uninfected skin levels. No differences were found between MS skin and its corresponding uninfected control (**Figure 8**). Immunoassays were performed to examine levels of TGFβ1 and IL-18 cytokines in supernatants. IL-18 levels were increased in supernatant samples from MSOA skin compared to the oleic acid uninfected control (73.9 pg/ml, IQR 25.1 to 135.1, vs. 8.5 pg/ml, IQR 5.8 to 11.4; *p*=0.03) (**Figure 8**). There were no differences in levels of TGFβ1 and IL-18 in supernatants from MS skin and uninfected control skin (**Figure 8**). TGFβ1 levels did not significantly change in supernatant samples, which contradicts what was observed in the gene expression analysis. This may be due to post-transcriptional regulation of TGFβ1. Addition of OA to uninoculated skin resulted in no significant changes in IL-18 and TGFβ1 levels as measured by RNA or protein levels.

### Nine Allergens of *M. sympodialis* Were Identified in Inoculated Skin

We next examined protein levels of *M. sympodialis* allergens when the yeast was inoculated onto the skin explant model and incubated for 6 days. A total of 202 *M. sympodialis* proteins were detected by mass spectrometry and identified by matching peptide fingerprints to *M. sympodialis* (reference strain ATCC 42132) protein sequences (Swiss-Prot database). However, after filtering to include proteins found only in *M. sympodialis* inoculated skin in ≥2 biological experiments, only 48 fungal proteins were included in the final analysis.

Most of the proteins were found only in MSOA skin (32/48, 66%); 12 (25%) were uncharacterized proteins, nine (19%) were allergens, and only one lipolytic enzyme, Lipase 3, was identified in MSOA skin. The allergens found in infected skin were Mala S1, Mala S4, Mala S5, Mala S6, Mala S8, Mala S9, Mala S11, Mala S12, Mala S13. Mala S11, and Mala S13 were the only two allergens found in both MSOA and MS skin (**Supplementary Material S2**).

## Discussion

We have characterized the host-pathogen interactions of *M. sympodialis* with human skin at the molecular level using a human skin explant model. The model consisted of inoculating skin explants with *M. sympodialis* yeasts and incubating them for 6 days. The influence of added exogenous oleic acid, mimicking lipid-rich skin niches, was also investigated. First, the presence of yeasts was confirmed by fluorescence microscopy and SEM in the inoculated samples. Then, host responses were evaluated by gene expression, proteomics and immunoassays.

In this model, increased mRNA and protein expression of β-defensin 3 and RNase 7 was detected in MS skin, similar to previous reports in skin of individuals with pityriasis versicolor and atopic dermatitis (Gambichler et al., 2008; Brasch et al., 2014). However, AMP gene expression was not detected when oleic acid was applied to the skin. Oleic acid was added to this model to allow evaluation of host response to *M. sympodialis* on oily skin. Previous studies found fatty acids (oleic acid, palmitic acid and lauric acid) to be protective, resulting in the upregulation of β-defensin 2 expression by sebocytes in cell culture (Nakatsuji et al., 2010). This is contrary to what was observed with this model in the non-inoculated and MSOA skin and may be due to the high concentration of OA used in the current report. Skin damage by OA has been documented previously with concentrations as low as 5% and is macroscopically evident as dermatitis in healthy volunteers and histological damage in reconstructed epidermis (Boelsma et al., 1996). Therefore, the lack of AMP expression in OA or MSOA skin may be due to epidermal damage as a result of OA supplementation, which requires further validation. The direct effect of OA on skin was analyzed here by proteomics and the majority of proteins (including keratins involved in cornification, keratinocyte differentiation and wound healing responses) were detected at lower levels in OA skin compared to untreated skin. Only a handful of proteins had increased levels in OA skin but the differences in fold change were not statistically significant. It is known that OA can damage and produce desquamation of the *stratum corneum* (DeAngelis et al., 2005), which was also observed in histological sections in our model. In addition, OA-induced damage facilitates penetration of *M. sympodialis* so that it contacts and damages keratinocytes in deeper skin layers. These two consequences could result in the absence of AMPs produced by the epidermis in MSOA skin.

The lack of AMP protein expression in MSOA skin in this model is similar to what has been reported in skin lesions of AE individuals, where previous studies have documented decreased or no expression of β-defensin 2 and LL37 in acute and chronic skin lesions of AE individuals (Ong et al., 2002; Clausen et al., 2018). Both AMP genes and protein expression in the epidermis have been reported to be lower in atopic dermatitis patients compared to psoriasis patients (de Jongh et al., 2005).

High levels of certain S100 proteins were found in MS skin (S100A2, S100A4, and S100B). These have not been reported to have a role in *Malassezia* infections or allergic reactions. However, these three S100 proteins have been reported to have a role in macrophage migration, cell proliferation and migration, and as apoptosis inhibitors and regulators of p53 protein (Donato et al., 2013). The increase of these S100 proteins should be confirmed with different techniques, such as gene expression, immunoassays, or immunofluorescence as LC-MS/MS could misidentify these peptides with other similar proteins sharing S100 domains such as filaggrin (Bunick et al., 2015). This differentiation will be important as filaggrin is essential for epidermal barrier formation (Hänel et al., 2013) and the loss of its function is already recognized as a causative factor for AE (Nutten, 2015). Further study of the role of filaggrin in the response to *M. sympodialis* is required. In addition, the lower expression of *S100A9* gene found in skin inoculated with *M. sympodialis* has not been reported before. The expression of *S100A9* gene was expected to be increased or unchanged as seen with the rest of S100 proteins. In order to investigate whether decreased expression of *S100A9* is a feature of *M. sympodialis* skin response, a follow up of the dynamics of *S100A9* gene and protein expression in earlier and later stages of *M. sympodialis* infection would be required.

Uninfected OA skin had significantly higher levels of TNFα, IL1-β, and IL-6 compared to MSOA. These higher cytokine levels may reflect an inflammatory effect of OA on skin, which as mentioned above is associated with skin damage.

The effect of *Malassezia* species on cytokine production can vary depending on the clinical and experimental context (Faergemann et al., 2001; Watanabe et al., 2001; Pedrosa et al., 2019). High levels of IL-1β, IL-6, IL-8, and TNFα have been found in supernatants of human keratinocyte cell cultures after 3–6 h of co-incubation (Watanabe et al., 2001). However, the cytokine response to *Malassezia* sp. varies depending on the species; *M. pachydermatis* induced the highest levels of all these cytokines and *M. furfur* induced almost no response (Watanabe et al., 2001). In our study, none of these cytokines had high levels when MS skin was compared to the corresponding OA non-inoculated controls and TNFα, IL1-β, and IL-6 levels were significantly decreased in MSOA skin. This finding can be explained in three ways. Firstly, the time point for cytokine analysis differs. In a recent study using reconstructed epidermis, gene expression of *IL1B, IGFB1*, and *TNFA* in tissue was increased after 6 h incubation with *M. sympodialis*, but these same genes were downregulated after 48 h of co-incubation (Pedrosa et al., 2019). Secondly, models to study host response to fungal infection can differ from the response in real human infections. Faergemann et al. (2001) reported no difference in skin TNFα levels in individuals with *Malassezia* folliculitis and seborrheic dermatitis when compared to healthy volunteers, similar to the current *ex vivo* skin model and contrary to what has been reported from monolayer keratinocyte culture. In addition, lower TNFα serum levels were found in individuals with chronic AE, along with low serum levels of IL-10, β-defensin 3 and high levels of β-defensin 2 (Kanda and Watanabe, 2012). Finally, the lack of AMP expression seen in the MSOA skin can explain the low levels of some cytokines as these AMPs (especially, β-defensins, S100 proteins and cathelicidin) are key for inducing cytokine responses (Niyonsaba et al., 2017).

Thomas et al. (2008) demonstrated that lack of the external lipid layer in *Malassezia* species increased the inflammatory response by keratinocytes, characterized by high levels of IL-6, IL-8, and IL-α. Similarly, Kesavan et al. (2000) reported that *Malassezia* cells with lipid-depleted surfaces triggered higher levels of inflammatory cytokines such as IL-1β, TNFα and IL-6. In our model of *M. sympodialis* infected skin, supplementation with oleic acid had the opposite effect, with reduced levels of acute inflammatory cytokines. The direct effect of oleic acid on *M. sympodialis* cells and the cell wall was not evaluated in this study, hence the impact of OA on cytokine responses to *M. sympodialis* cannot be considered; however, it is a factor currently under investigation by our group.

*IL18* and *TGFB1* were highly expressed in MSOA skin, whilst only IL-18 levels were significantly higher in the analyzed supernatants. IL-18 belongs to the IL-1 cytokine family, along with IL-1α and IL-1β, and is cleaved by caspase-1 after being activated by 3NLRP inflammasome (Fenini et al., 2017). This inflammasome pathway is crucial for inducing Th1/Th2 responses. IL-18 induces the formation of high serum levels of IgM, IgG_1_, IgG_2a,_ and very high levels of IgE antibodies (Enoksson et al., 2011). The production of these antibodies depends on CD4^+^ T cell-derived IL-4 and the auto-reactivity of these antibodies is regulated and depends on NK T cells (Enoksson et al., 2011). The production of high levels of IL-18, along with high levels of IL-4 (Th2 biased response), has been associated with worse prognosis in other infections such as leishmaniasis (Gurung et al., 2015). High serum levels of IL-18, IL12/p40 and IgE antibodies have been found in individuals with atopic eczema and their serum levels correlate proportionally with clinical severity of AE skin lesions (Zedan et al., 2015).

As mentioned previously, *M. sympodialis* allergens play a crucial role in the pathogenesis of atopic dermatitis (Gioti et al., 2013). In this study, higher numbers of allergens were identified in the MSOA skin compared to MS skin. This finding could be explained by the higher number of yeasts present on the surface of MSOA skin. Due to the skin damage caused by OA, it is possible that these allergens were in contact with inner epidermal cells and contributed to the host response seen in MSOA skin. The role of allergens in *M. sympodialis* pathogenicity is a future avenue of investigation with this *ex vivo* human skin model.

In conclusion, the local host response to *M. sympodialis* can be characterized using this *ex vivo* human skin model. Such host response can vary as previously described, depending on the *Malassezia* species, host intrinsic and extrinsic factors, time of clinical evolution, and type of infection. Most of these conditions can potentially be mimicked in this *ex vivo* skin model. Comparison of responses between different skin conditions has already been shown to be possible in this study. In non-oily and intact skin, AMPs and S100 proteins are key in the response to *M. sympodialis*, but in oily and damaged skin, allergens and yeasts are in direct contact with keratinocytes, and inflammasome responses seem to lead to increased IL-18, which can promote chronic inflammation, auto-reactivity in skin and continuing local damage.

## Data Availability Statement

The datasets presented in this study can be found in online repositories. The names of the repository/repositories and accession number(s) can be found in the article/**Supplementary Material**.

## Author Contributions

DC, DM, and CM conceptualized and designed the study. DC performed the experiments and data analysis. All authors contributed to the article and approved the submitted version.

## Funding 

This project was funded by a Wellcome Trust Strategic Award for Medical Mycology and Fungal Immunology (097377/Z/11/Z). We would like to acknowledge the support of Internal Funding through a Core Facilities Voucher from the University of Aberdeen.

## Conflict of Interest

The authors declare that the research was conducted in the absence of any commercial or financial relationships that could be construed as a potential conflict of interest.

## Supplementary Material

S1 Results **Supplementary Material S1** proteomics human database OA.

S2 Results **Supplementary Material S2** *Malassezia sympodialis* proteins.

S3 Results **Supplementary Material S3** Analysis of uninfected skin samples with and without OA.
